# Complexities, Benefits, Risks, and Clinical Implications of Sodium Bicarbonate Administration in Critically Ill Patients: A State-of-the-Art Review

**DOI:** 10.3390/jcm13247822

**Published:** 2024-12-21

**Authors:** Akram M. Eraky, Yashwanth Yerramalla, Adnan Khan, Yasser Mokhtar, Alisha Wright, Walaa Alsabbagh, Kevin Franco Valle, Mina Haleem, Kyle Kennedy, Chad Boulware

**Affiliations:** 1 Emergency Medicine, Freeman Health System, Joplin, MO 64804, USA; drwright2b@hotmail.com (A.W.); kwkennedy@me.com (K.K.); drbware78@gmail.com (C.B.); 2 Graduate Medical Education, Kansas City University, Kansas City, MO 64106, USA; 3 Pulmonology and Critical Care Medicine, Freeman Health System, Joplin, MO 64804, USA; yerray02@gmail.com (Y.Y.); akhan3@freemanhealth.com (A.K.); ymmokhtar@freemanhealth.com (Y.M.); 4 Internal Medicine, Northern General Hospital, Sheffield S5 7AU, UK; walaa.alsabbagh1@nhs.net; 5 Anesthesiology Department, University of Michigan Medical School, University of Michigan, Ann Arbor, MI 48109, USA; kevin.f92@hotmail.com; 6 Nephrology Unit, Department of Clinical and Experimental Internal Medicine, Medical Research Institute, Alexandria University, Alexandria 5422031, Egypt; minahhyoussef@gmail.com

**Keywords:** sodium bicarbonate, metabolic acidosis, acid–base balance, diabetic ketoacidosis, lactic acidosis, rhabdomyolysis, high anion gap, normal anion gap, acute kidney injury, cardiac arrest, resuscitation, cerebrospinal fluid acidosis

## Abstract

Sodium bicarbonate has been used in the treatment of different pathologies, such as hyperkalemia, cardiac arrest, tricyclic antidepressant toxicity, aspirin toxicity, acute acidosis, lactic acidosis, diabetic ketoacidosis, rhabdomyolysis, and adrenergic receptors’ resistance to catecholamine in patients with shock. An ongoing debate about bicarbonate’s efficacy and potential harm has been raised for decades because of the lack of evidence supporting its potential efficacy. Despite the guidelines’ restrictions, sodium bicarbonate has been overused in clinical practice. The overuse of sodium bicarbonate could be because of the desire to correct the arterial blood gas parameters rapidly instead of achieving homeostasis by treating the cause of the metabolic acidosis. Moreover, it is believed that sodium bicarbonate may reverse acidosis-induced myocardial depression, hemodynamic instability, ventricular arrhythmias, impaired cellular energy production, resistance to catecholamines, altered metabolism, enzyme suppression, immune dysfunction, and ineffective oxygen delivery. On the other hand, it is crucial to pay attention to the potential harm that could be caused by excessive sodium bicarbonate administration. Sodium bicarbonate may cause paradoxical respiratory acidosis, intracellular acidosis, hypokalemia, hypocalcemia, alkalosis, impaired oxygen delivery, cerebrospinal fluid acidosis, and neurologic dysfunction. In this review, we discuss the pathophysiology of sodium bicarbonate-induced adverse effects and potential benefits. We also review the most recent clinical trials, observational studies, and guidelines discussing the use of sodium bicarbonate in different pathologies.

## 1. Introduction

Sodium bicarbonate is commonly used in clinical practice to treat different pathologies, such as hyperkalemia, cardiac arrest, and toxic ingestion of tricyclic antidepressants and aspirin. Moreover, sodium bicarbonate has been used in the treatment of different types of acute metabolic acidosis, such as lactic acidosis (LA), diabetic ketoacidosis (DKA), and rhabdomyolysis. However, the evidence of its efficacy in the treatment of these different pathologies varies significantly, leading to decades of ongoing debate regarding its benefits and potential harm [1,2,3,4,5].

The goal of the management of the previously mentioned pathologies is to restore homeostasis and provide a suitable environment for enzymes’ functionality and biochemical reactions in different body organs. One of the medications that are commonly used to achieve homeostasis is sodium bicarbonate because of its theoretical ability to correct blood pH rapidly, subsequently reversing the adverse effects induced by acute acidosis, such as myocardial depression, hemodynamic instability, ventricular arrhythmias, impaired cellular energy production, decreased responsiveness to catecholamines, altered metabolism, enzymes suppression, immune dysfunction, coagulopathy, and ineffective oxygen delivery [6,7,8].

## 2. Discussion

There are two approaches to treating acute acidosis. The first is a gradual method that focuses on addressing the underlying causes of acidosis. The second approach involves administering a buffering agent, such as intravenous sodium bicarbonate, in addition to treating the root cause to rapidly normalize arterial blood gas (ABG) parameters. Treating acute acidosis with sodium bicarbonate may appear advantageous; however, sodium bicarbonate administration in acidotic patients may result in harm. Sodium bicarbonate was found to be associated with many adverse effects, such as impaired oxygen delivery, cerebral edema, paradoxical cerebrospinal fluid (CSF) acidosis, neurologic dysfunction, hypocalcemia, hypokalemia, increased arterial partial pressure of carbon dioxide (PCO_2_), lactate production, and paradoxical intracellular acidosis [9,10,11,12,13].

Herein, we discuss the effects of sodium bicarbonate on overall mortality, CSF pH, neurological status, cerebral edema, and oxygen delivery. We also highlight the potential pathophysiology of sodium bicarbonate-induced neurological dysfunction. Moreover, this review provides insight into the effects of sodium bicarbonate in special subgroups, such as patients with acute kidney injury (AKI), Rhabdomyolysis, DKA, LA, normal anion gap metabolic acidosis (NAGMA), cardiac arrest, and hyperkalemia. Additionally, we mention the most recent clinical trials, observation studies, and guidelines regarding the use of sodium bicarbonate in different situations.

### 2.1. The Effect of Sodium Bicarbonate Administration on the Neurological Status

A few studies have investigated the association between sodium bicarbonate administration and neurological outcomes in adults. In patients with cardiac arrest, prehospital administration of sodium bicarbonate was negatively associated with neurological outcomes to hospital discharge [14]. In a randomized clinical trial by Touron et al., prehospital administration of sodium bicarbonate was significantly associated with worse neurologic outcomes in the North American population compared to the control group [15]. In another retrospective study by Zaidi et al., sodium bicarbonate administration was found to be significantly associated with the reduction in the neurological recovery at 30 days compared to the control group [16].

This neurological dysfunction, which is associated with sodium bicarbonate administration, could be explained by the bicarbonate-induced changes in arteriolar PCO_2_ and HCO_3_. These changes create pH disequilibrium between CSF and plasma, subsequently leading to acute paradoxical CSF acidosis and neurologic dysfunction, as discussed below.

#### 2.1.1. The Effect of Sodium Bicarbonate Administration on the CSF pH

The CSF is one of the main components of the intracranial space besides the brain parenchyma and blood. The CSF is secreted by the choroid plexus, which is a highly vascular tissue in the ventricles, at a rate of 0.3 to 0.4 cc per minute; then, the CSF flows from the ventricular system into the subarachnoid space, where the arachnoid villi reabsorbs the CSF into the dural sinuses [17,18,19].

According to Stewart’s approach, three variables regulate and affect the pH of biological fluids. These factors include (1) PCO_2_, (2) the concentration difference between strong anions and cations (SID), and (3) the weak nonvolatile buffers, such as albumin and phosphate. In contrast, CSF does not contain nonvolatile buffers, such as albumin and phosphate. Consequently, the pH of CSF is controlled by PCO_2_ and SID. Since the CSF does not have nonvolatile weak acid buffers, SID in the CSF is identical to bicarbonate concentration [20,21,22]. As a result, the carbon dioxide–bicarbonate ratio is almost the only factor that affects the CSF pH [20,21,22,23].

The CSF pH plays an important role in controlling breathing in response to acute acidemia. In previous animal and clinical studies, ventilation varied in response to CSF pH manipulation to restore normal CSF pH. During CSF acidification, the ventilation rate increased. In contrast, the ventilation rate decreased during CSF alkalinization [20,22,24,25,26,27]. In patients with a subarachnoid hemorrhage, increased lactate concentration in the CSF results in a reduction in the CSF SID. This localized low pH in the CSF results in stimulation of the respiratory rate that causes compensatory CSF hypocapnia that results in a normal CSF acid–base balance. Simultaneously, this reflex causes arterial respiratory alkalosis [27]. This illustrates the bidirectional relationship between arterial PCO_2_ and CSF pH, where both can influence each other.

The blood–brain barriers (BBBs), including both the blood–CSF and blood–brain barrier, are impermeable to most polar substances and ions. In contrast, it is freely permeable to CO_2_ [20,28,29,30]. Subsequently, changes in arterial PCO_2_ can affect CSF pH dramatically in patients with respiratory acidosis or those receiving sodium bicarbonate, while increased acid production in patients with metabolic acidosis does not affect CSF pH because of the protective role of BBB [9]. We see this strong effect of arterial CO_2_ on the neurological status in the daily practice of medicine. Patients with acute respiratory acidosis usually develop neurologic dysfunction rapidly and may develop lethargy and coma, while patients with severe acute metabolic acidosis can still be awake, alert, and oriented. The explanation for this phenomenon is that CO_2_ moves easily through the BBB and changes CSF pH rapidly. In contrast, the BBB prevents protons and acids from passing to the CSF in patients with metabolic acidosis, subsequently protecting CSF pH and keeping it near normal [9,23,31]. This explains how human body can tolerate severe metabolic acidosis by protecting CSF pH through the protective role of the BBB.

**Figure 1:**
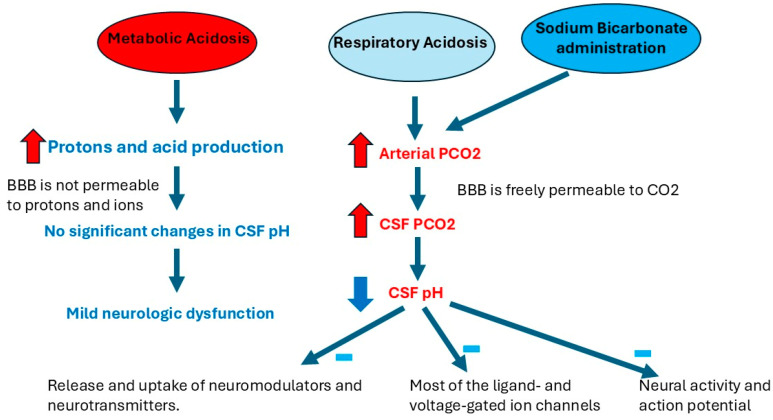
Pathophysiology of developing neurological dysfunction in patients with acute respiratory acidosis and after sodium bicarbonate administration. Sodium bicarbonate administration causes acute increase in arterial PCO_2_. Since BBB is freely permeable to CO_2_, high arterial PCO_2_ causes CSF acidosis. CSF acidosis affects the release and uptake of neuromodulators and neurotransmitters and the activity of ion channels. Subsequently, this affects neural activity and the action potential. In contrast, acute metabolic acidosis causes mild changes in CSF pH; as a result, it causes mild neurological dysfunction. Abbreviations: PCO_2_, partial pressure of carbon dioxide; CSF, cerebrospinal fluid; BBB, blood–brain barrier.

This previous explanation was assessed by Posner et al., who compared CSF pH and arterial pH in patients with severe metabolic acidosis to those with respiratory acidosis. Of interest, Posner et al. found that CSF pH values were near normal and the mental status was intact in patients with severe metabolic acidosis [9,23]. In contrast, patients with acute respiratory acidosis were found to have severe CSF acidosis and subsequently developed neurological dysfunction, stupor, and coma because of the high diffusibility of CO_2_ through the BBB [9,32]. These previous studies demonstrate that low CSF pH and increased arterial and CSF PCO_2_ are associated with declining neurological status, while arterial pH is not associated with neurological dysfunction. It also shows that the CSF pH is near normal in patients with metabolic acidosis because BBB is impermeable to the high levels of the newly produced acids in the blood, while patients with respiratory acidosis have severe acidotic CSF because of the high diffusibility of CO_2_ through the BBB. This may explain why individuals with severe metabolic acidosis may have a lower arterial pH but maintain normal neurological status. On the other hand, patients with respiratory acidosis may have a less acidic arterial pH but experience greater neurological dysfunction (Figure 1).

Theoretically, bicarbonate administration may increase both PCO_2_ and serum bicarbonate levels. However, CO_2_ movement across the BBB is faster than bicarbonate. This may cause paradoxical CSF acidosis, resulting in neurological dysfunction [31]. This may explain why administering sodium bicarbonate is associated with neurologic dysfunction in the previously discussed studies [9,31] (Figure 1).

#### 2.1.2. The Effect of CSF pH on the Extracellular Interstitial Fluid (IF) in the Brain

**Figure 2:**
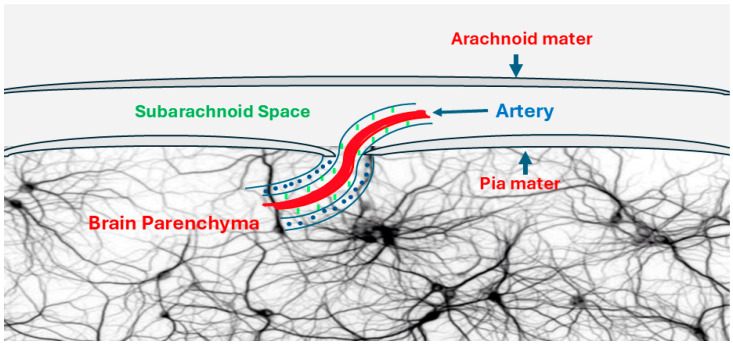
The Virchow–Robin Space surrounding a cortical artery. Virchow–Robin Space (green-dotted area) is a perivascular space surrounding arteries, arterioles, venules, and veins. Virchow–Robin Space passes to the brain parenchyma through the subarachnoid space and the subpial space (blue-dotted area). Virchow–Robin Space connects the CSF in the subarachnoid space to the IF in the brain parenchyma.

The extracellular fluid surrounding brain cells and neurons is connected to the CSF in the subarachnoid space through the glymphatic system. CSF is produced by the choroid plexus in the ventricular system and then flows through the ventricular system into the subarachnoid space. In the subarachnoid space, the CSF flows through the periarteriolar space into the interstitial fluid (IF) surrounding the brain neurons and cells. This periarteriolar space is created by astrocytic footplates. The Aquaporin-4 channels (AQP-4) on glial footplates help IF and the CSF to mix. Then, the IF drains into the dural sinuses through the perivenous spaces [33,34,35,36] (Figure 2).

This perivascular space surrounding arteries, arterioles, veins, and venules is known as the Virchow–Robin space. The Virchow–Robin space passes through the subarachnoid and subpial spaces to the brain parenchyma to transport the CSF from the subarachnoid space to the brain parenchyma and back from the IF to the venous sinuses [33,34,35,36,37] (Figure 2). This shows that any changes in the CSF pH or pressure directly affect the IF pH and pressure.

Changes in the pH of the extracellular IF can affect brain functionality. Low IF pH affects both the ligand- and voltage-gated channels. As a result, any changes in the IF pH affect neural activity. In general, a decrease in the IF pH inhibits neural activity and most of ion channels, while the increase in the IF pH stimulates neural activity. This pH-induced neural activity is usually associated with an increase in the activity of the N-methyl-D-aspartate receptors (NMDA) [38,39].

Changes in the IF pH can also affect the uptake and release of both the neuromodulators and neurotransmitters. For instance, acidosis of the synaptic cleft was found to have an inhibitory effect on the presynaptic voltage-gated Ca^2+^ channels [40,41]. Moreover, extracellular acidosis was found to decrease the vesicular release of glutamate in rat brain synaptosomes, while the uptake of glutamate did not change. In contrast, the uptake of the acetylcholine precursor, choline, was also found to be inhibited in low extracellular pH [42]. In another study, both low extracellular and intraterminal pH stimulated the release of tritiated dopamine [43]. In summary, changes in the extracellular IF pH affect (1) the ligand- and voltage-gated ion channels in the neural cells, (2) neural activity, (3) action potential, and (4) the release and uptake of neuromodulators and neurotransmitters. This may explain why patients with acidotic CSF and IF develop neurological dysfunction (Figure 1).

#### 2.1.3. Sodium Bicarbonate-Induced Cerebral Edema

Some animal and clinical studies have reported an association between sodium bicarbonate administration and the development of cerebral edema [31,44,45,46]. Nevertheless, these clinical studies discussed the association between sodium bicarbonate and cerebral edema only in children and young adolescents. No clinical studies investigated this association in adults [4]. Clinical studies are encouraged to investigate the association between sodium bicarbonate administration and developing cerebral edema in adults.

One potential pathophysiology of developing cerebral edema following sodium bicarbonate administration is sodium bicarbonate-induced cerebral hypoxia. In an animal study by Bureau et al., sodium bicarbonate administration significantly decreased the partial pressure of oxygen in the cerebrospinal fluid (PcsfO_2_) in both the HCl-acidotic dogs and the ketoacidotic dogs [47]. In contrast, no significant changes in the PcsfO_2_ were noticed in the ketoacidotic dogs that were not treated with sodium bicarbonate. A negative association was also noticed between the cisternal PO_2_ and the cisternal lactic acid [47]. This animal study shows that sodium bicarbonate does not only affect oxygen delivery in the bloodstream, but also in the cerebral blood and CSF. This hypoxia may induce cellular injury in the brain, leading to the development of cerebral edema.

Another potential mechanism by which sodium bicarbonate may induce cerebral edema is increasing cerebral blood flow (CBF). Paradoxical CSF acidosis after bicarbonate administration could induce arterial vasodilatation, leading to increased CBF and cerebral edema. In a study by Nakashima et al., the administration of sodium bicarbonate caused a significant increase in the mean CBF in all areas of the brain. They also found that sodium bicarbonate is associated with decreased intracellular pH in the whole body [48]. This increased CBF after sodium bicarbonate administration may explain the incidence of cerebral edema after the use of sodium bicarbonate.

### 2.2. The Effect of Sodium Bicarbonate Administration on Tissue Oxygenation

Hemoglobin’s affinity to oxygen varies according to many factors, such as the pH and the PCO_2_. This variance in hemoglobin affinity makes it suitable for its physiological role. Sodium bicarbonate administration can affect hemoglobin’s affinity to oxygen in different ways. Rapid administration of sodium bicarbonate may cause a rapid increase in pH, subsequently inducing alkalosis. Alkalosis increases hemoglobin’s affinity to oxygen and may cause a left shift in the oxygen–hemoglobin dissociation curve. In an animal study by Douglas et al., sodium bicarbonate administration led to an increase in arterial oxygen tensions and arterial oxyhemoglobin saturation and a decrease in venous oxygen tension. This indicates that sodium bicarbonate increases hemoglobin’s affinity to oxygen, which leads to increased arterial oxyhemoglobin saturation and decreased venous PO_2_. This indicates that sodium bicarbonate may cause a left shift in the oxygen–hemoglobin dissociation curve, which could impair peripheral oxygen delivery [49].

Sodium bicarbonate may also cause acute paradoxical respiratory acidosis by increasing PCO_2_, particularly in patients with lung disease who cannot get rid of the excess CO_2_ created by sodium bicarbonate administration. At the pulmonary vasculature level, increased PCO_2_ may decrease the hemoglobin affinity to oxygen and cause a right shift in the oxygen–hemoglobin dissociation curve. This may affect the oxygenation of the peripheral tissues [50,51]. We suggest that when the sodium bicarbonate-induced PCO_2_ is high enough to cause paradoxical respiratory acidosis, it may cause a right shift in the oxygen–hemoglobin dissociation curve, especially in patients with lung disease.

In a study by Munk et al., there was no significant association between sodium bicarbonate administration and changes in the oxygen–hemoglobin dissociation curve in children with diabetic ketoacidosis. This indicates that sodium bicarbonate-induced changes in the oxygen–hemoglobin dissociation curve were not significant enough to change tissue oxygenation [52]. In another study by Battaglia et al., sodium bicarbonate administration was found not to be associated with significant changes in the oxygen–hemoglobin dissociation curve [53]. These two studies doubt the significant influence of sodium bicarbonate on the oxygen delivery. This could be because of the heterogeneous population sample, as many factors can alter the effect of sodium bicarbonate on the oxygen–hemoglobin dissociation curve, such as the presence of lung disease, sodium bicarbonate dose, and speed of bicarbonate administration. More studies are encouraged to assess the effect of sodium bicarbonate on the oxygen–hemoglobin dissociation curve. Studying the effect of metabolism manipulators of sodium bicarbonate, such as carbonic anhydrase inhibitors, is encouraged also.

### 2.3. The Effect of Sodium Bicarbonate on Intracellular Acidosis and Lactic Acid Production

Sodium bicarbonate may cause paradoxical intracellular acidosis. Bicarbonate binding to hydrogen ions in the extracellular fluid decreases hydrogen ions concentration and increases the pH extracellularly. This reaction between bicarbonate ions and hydrogen ions results in carbonic acid formation and subsequently, CO_2_ production. CO_2_ can diffuse across cell membranes and combine with H_2_O intracellularly to form carbonic acid, which decreases pH intracellularly [54,55].

In an in vitro study by Ritter et al., sodium bicarbonate administration was found to cause a rapid increase in the extracellular pH with simultaneous intracellular acidosis in human platelets that are suspended in a Tyrode’s buffer [54]. Of interest, acetazolamide, a carbonic anhydrase inhibitor, has been shown to suppress sodium bicarbonate-induced intracellular acidosis [54]. In another in vitro study using suspensions of leucocytes, lactic acid and propionic acid were added extracellularly to mimic metabolic acidosis environment. Sodium bicarbonate was found to cause intracellular acidosis. However, this effect was transient with the use of a series of small boluses of sodium bicarbonate [55].

Theoretically, increased intracellular acidosis may increase the production of lactic acid. Of interest, serum levels of lactic acid were noticed to increase after sodium bicarbonate administration [56,57,58]. The role of acetazolamide in offsetting the intracellular acidosis induced by bicarbonate is promising, as shown in previous studies [54]. More clinical studies are encouraged to evaluate the effect of simultaneous administration of carbonic anhydrase inhibitors with sodium bicarbonate on mortality.

### 2.4. The Effect of Sodium Bicarbonate on Adrenergic Receptors and Hemodynamic Status

It is generally believed that acidosis has negative consequences on the expression of adrenergic receptors and their sensitivity. Regarding beta receptors, many experimental studies reported a decrease in both the sensitivity and expression of beta receptors, subsequently declining myocardial contractility in response to acidosis [59,60,61,62,63,64]. However, an experimental study by Schotola et al. showed that there is no difference in contractility and catecholamine response between mild acidosis and normal pH [65]. In another study by Andersen et al., cardiac output decline started when the pH was less than 6.9 [66]. These two studies suggest that the negative myocardial effects of acidosis are most likely linked to its more severe forms [65,66]. Besides its effect on beta-receptors, acidosis might also impact contractility negatively through inhibiting the responsiveness of myofibrils to calcium ions [67].

Regarding alpha receptors, acidosis was found to attenuate alpha 1-mediated vasoconstriction by phenylephrine and potassium chloride in human skeletal muscle arteries [68]. In accordance with this study, many other in vivo studies showed that acidosis may have a negative impact on arterial vasoconstriction, especially medium-sized arteries [69,70,71]. In contrast, acidosis was found to be associated with increased vasoconstriction in some in vitro studies [72,73,74,75]. These different effects of acidosis on arterial vasoconstriction or dilatation may be due to the varying expression of different adrenergic receptors in different sizes of blood vessels and collecting the arterial samples from different species. More in vivo and in vitro studies are encouraged to understand the effect of acidosis on the vasoactive properties of blood vessels.

#### Sodium Bicarbonate in Acidotic Patients Treated with Vasopressors

Theatrically, acidotic patients with hypotension may benefit from sodium bicarbonate, as acidosis affects the sensitivity of adrenergic receptors to vasopressors and myocardial contractility, as discussed above. Correcting acute acidosis in those patients with administration of sodium bicarbonate is theoretically reasonable to increase the adrenergic receptor’s sensitivity to vasopressors and reverse acidosis-induced myocardial dysfunction. In two clinical trials, sodium bicarbonate did not affect hemodynamics significantly in patients with metabolic acidosis compared to the normal saline group. These two clinical trials are small-sized. One of them had ten patients, and the other had fourteen patients [10,76]. In a larger clinical study by Fang et al., the effects of different types of fluids on the hemodynamics were evaluated in septic patients who did not receive vasopressors during the initial two hours [77]. They found that there is no difference in mean arterial pressure (MAP), cardiac output, and heart rate between normal saline, sodium bicarbonate, and hypertonic saline. However, sodium bicarbonate was found to improve the MAP more rapidly than normal saline and hypertonic saline [77].

Fujii et al. found that early sodium bicarbonate administration in vasopressor-dependent patients with metabolic acidosis was associated with higher MAP at 6 h with odds ratio of 5.99 (95% CI, from 1.84 to 10.2) and higher Delta MAP per vasopressor dose at 6 h with odds ratio of 8.87 (95% CI, from 3.34 to 14.48) compared to those who did not receive sodium bicarbonate. This indicates that sodium bicarbonate could be associated with higher responsiveness to vasopressors in acidotic patients who are dependent on vasopressors [78]. However, there was no statistical difference in intensive care unit (ICU) mortality. This might be because patients who received early sodium bicarbonate were sicker and had lower pH and higher lactate levels at the time of metabolic acidosis diagnosis [78].

### 2.5. The Effect of Sodium Bicarbonate Administration on Mortality in Acidotic Patients

The administration of sodium bicarbonate in patients with acute acidosis is compelling, as it theoretically facilitates the rapid normalization of ABG parameters, subsequently correcting the patient’s acidosis and offsetting the acidosis-induced adverse effects. However, sodium bicarbonate administration may have many side effects, such as hypernatremia, hypocalcemia, decreased tissue oxygenation, and CO_2_ retention, especially in patients with lung disease. The retention of CO_2_ may cause paradoxical respiratory acidosis, subsequently increasing the intracellular acidosis [31,79]. Moreover, sodium bicarbonate administration could also cause paradoxical CSF acidosis and altered mental status [9,23,31].

In a systematic review published in 2019 by Fujii et al., they found twelve studies discussing the physiological and biochemical effects of sodium bicarbonate and only two studies discussing the clinical effects in acidotic patients [80]. One of the two studies, which discussed the clinical effects, compared sodium bicarbonate and Tris hydroxymethyl aminomethane (THAM) in 18 ICU patients with mild metabolic acidosis. The study found that both THAM and sodium bicarbonate produced a similar alkalinizing effect. Of interest, sodium bicarbonate was found to decrease potassium levels and increase sodium levels compared to THAM [81]. Hoste et al. did not report other clinical outcomes, such as ICU length of stay, mortality rate, and MAP changes [81]. The other study by Jaber et al. studied the use of sodium bicarbonate in acidotic patients with a pH of 7.15 (7.09, 7.18). Jaber et al. found no statistically significant difference in 28-day mortality or the development of organ failure by day 7 between the sodium bicarbonate group with a pH of 7.15 (7.09–7.18) and the control group with a pH of 7.15 (7.11, 7.18) [82]. They also observed more episodes of hypernatremia, hypocalcemia, and metabolic alkalosis in patients treated with sodium bicarbonate [82].

In an international retrospective study by Fujii et al. in 2021, there was no statistical difference in-hospital mortality, ICU mortality, delta MAP per vasopressor dose at 6 h and 12 h and vasopressor dose at 6 h and 24 h between acidotic patients with pH of 7.19 (7.11, 7.25) who received early sodium bicarbonate compared to acidotic patients with pH of 7.26 (7.21, 7.28) who did not receive bicarbonate in the overall population of the study [78]. The previously discussed studies indicate that there is no statistically significant difference in the overall mortality between sodium bicarbonate group and control. However, this might be because of the absence of subgrouping patients based on the different causes of acidosis and different co-morbidities. In this review, we discuss the use of sodium bicarbonate in various subgroups of acidotic patients, as well as its role in cardiac arrest resuscitation, AKI, and hyperkalemia in the upcoming sections.

#### 2.5.1. Sodium Bicarbonate in Patients with High Anion Gap Metabolic Acidosis

Metabolic acidosis is a common disorder frequently observed in the ICU. It may happen because of bicarbonate loss, resulting in NAGMA or overproduction of acid, leading to high-anion gap metabolic acidosis (HAGMA). An anion gap is calculated by subtracting the sum of chloride and bicarbonate concentrations from sodium concentration. All of these concentrations are measured in mEq/L, with a normal anion gap typically ranging from 8 mEq/L to 12 mEq/L. The high anion gap results from increased levels of unmeasured organic acids [83,84,85]. The causes of HAGMA include LA, DKA, pyroglutamic acid due to chronic use of acetaminophen, metformin, methanol, ethylene glycol, propylene glycol, and aspirin [83,84,85].

The calculated sodium level, not the corrected sodium level, is used to calculate the anion gap because, in hyperglycemic patients, the water shift, driven by the hyperosmolality to extracellular space, dilutes all electrolytes, including sodium, chloride, and bicarbonate, to a similar level. As a result, there is no need to correct electrolyte values in hyperglycemic patients to calculate the anion gap [85]. The anion gap should be corrected in the case of having low or high albumin. Albumin has a negative charge and represents the largest portion of normal anion gaps. As a result, any changes in albumin levels affect the anion gap. Since each gram of albumin has 2.5 mEq of negative charge, a value of 2.5 should be added to the anion gap for each 1 g/dL of albumin less than 4.5 g/dL. The following equation can be used to calculate the corrected anion gap. A corrected anion gap equals the sum of uncorrected anion gap and 2.5 × (4.5 − [Albumin]) [85,86]. Of interest, a corrected anion gap was found to be a potential predictor for the in-hospital mortality in patients with ischemic stroke and patients with sepsis-associated AKI [87,88].

##### Sodium Bicarbonate in Patients with DKA

Hyperglycemic emergencies, including DKA and hyperglycemic hyperosmolar state (HHS), are common emergencies and are the causes of frequent hospitalizations. Instead of treating the etiology of the acidosis and giving time for the pH to correct slowly, the rapid correction of pH and serum level of bicarbonate by sodium bicarbonate is appealing. However, sodium bicarbonate may be harmful and lead to many potential complications, such as paradoxical CSF acidosis, decreased oxygen delivery, hypocalcemia, hypokalemia, hypernatremia, hypercapnia, paradoxical respiratory acidosis, and intracellular acidosis [13,48,89,90,91]. Moreover, there is no clinical evidence to support the use of sodium bicarbonate routinely in patients with DKA [31].

Many clinical studies have shown that there is no association between sodium bicarbonate administration and mortality or time to acidemia resolution in patients with DKA and severe metabolic acidosis [31,92,93,94]. Additionally, Hale et al. found that sodium bicarbonate was associated with a lower fall in total blood ketone bodies and a delay in the fall in the serum lactate and lactate: pyruvate ratio compared to those who did not receive sodium bicarbonate [95]. The clinical and animal studies by Okuda et al. showed similar results [96]. The studies by Okuda et al. also show that sodium bicarbonate may have a stimulatory effect on hepatic ketogenesis and may increase the production of ketone bodies [96].

Regarding DKA patients with pH < 7.0, Duhon et al. found that neither time to hospital discharge nor time to resolution of acidosis was associated with intravenous bicarbonate [97]. The same results were found in a subgroup of patients with pH < 6.9. Of interest, the bicarbonate group required more fluids and insulin in the first 24 h [97]. Subgrouping DKA patients based on the presence of other pathologies such as AKI, concurrent NAGMA, concurrent lactic acidosis, and hyperkalemia is encouraged in further large-sized clinical trials and observational studies to evaluate the effect of sodium bicarbonate in these different subgroups.

The American Diabetes Association (ADA) guidelines in 2009, as well as its last update in 2024, state that it is sufficient to use intravenous fluids and insulin to treat the metabolic acidosis in patients with DKA and sodium bicarbonate is not recommended routinely because of the potential undesired consequences of using sodium bicarbonate. They also recommend considering sodium bicarbonate administration in severe metabolic acidosis with pH < 7.0 because of the adverse effects of severe acidosis. In this case, 100 mmol of 8.4% sodium bicarbonate solution in 400 mL of sterile water is recommended every two hours to achieve a goal of pH > 7.0 [2,98].

The guidelines by the Joint British Diabetes Societies for Inpatient Care in 2022 recommend resolving the metabolic acidosis in patients with DKA by intravenous fluid and insulin. They state that the use of sodium bicarbonate is not recommended, as bicarbonate may increase the partial pressure of CO_2_, subsequently causing paradoxical CSF acidosis. Sodium bicarbonate may also impair oxygen delivery and cause a delay in the fall in blood lactate: pyruvate ratio and ketones compared to normal saline [99]. Nevertheless, the 2022 guidelines by the Joint British Diabetes Societies for Inpatient Care suggest the occasional use of sodium bicarbonate in DKA patients when inotropes are required or if the pH remains low [99].

##### Sodium Bicarbonate in Patients with Rhabdomyolysis

Rhabdomyolysis results from traumatic and non-traumatic damage of the muscular tissue and release of the intracellular electrolytes and enzymes into the blood stream. Rhabdomyolysis may happen due to seizures, neuroleptic malignant syndrome, malignant hyperthermia, heat stroke, alcohol intake, infections, and electrolyte disturbance. It may also happen after exposure to some medications or toxins, such as statins, fibrates, antipsychotics, antidepressants, lithium, amphotericin B, antihistamines, cocaine, amphetamine, and heroin. The release of the intracellular content may result in many biochemical changes, such as an increase in the serum levels of creatinine kinase (CK), myoglobin, potassium, phosphorus, uric acid, lactate dehydrogenase, and creatinine, and a decrease in the serum levels of calcium. Patients with rhabdomyolysis may also develop HAGMA [100,101,102].

AKI is common in patients with rhabdomyolysis due to tubular blockage by myoglobin and intravascular hypovolemia due to fluid accumulation in the injured muscles. Moreover, the activation of multiple compensating mechanisms, such as antidiuretic hormone, the renin–angiotensin system, and the sympathetic nervous system may cause renal vasoconstriction, subsequently worsening the kidney injury. The main treatment of rhabdomyolysis, after removing the offending agent if exists, is fluid resuscitation. Fluid resuscitation decreases the ischemic injury to the kidney, enhances renal perfusion, and washes out the myoglobin precipitates in the tubules [4,100].

The use of sodium bicarbonate in patients with rhabdomyolysis for urine alkalinization is controversial. Theoretically, urine alkalinization by giving bicarbonate may attenuate the acidosis-induced renal vasoconstriction, production of free radicals, and myoglobulin precipitation in renal tubules. However, there is no clinical evidence supporting the use of sodium bicarbonate in patients with rhabdomyolysis [4,100,103,104,105].

In 2019, The Danish Society of Intensive Care Medicine (DSIT) and the Danish Society of Anesthesia and Intensive Care Medicine (DASAIM) suggested against the routine use of sodium bicarbonate for alkalization in patients with rhabdomyolysis to prevent AKI development; this was a weak recommendation with a low quality of evidence. Moreover, they mentioned that alkalinization may be considered in patients with rhabdomyolysis and severe acidosis [106]. The Scandinavian Society of Anesthesiology and Intensive Care Medicine (SSAI) Clinical practice committee (CPC) reviewed and supported the guidelines by DASAIM/DSIT on the prevention of AKI in patients with rhabdomyolysis [107].

In 2022, a recent retrospective study by Kim et al. evaluated the use of sodium bicarbonate in patients with rhabdomyolysis. Sodium bicarbonate was associated with a higher incidence of AKI compared to those who did not receive bicarbonate with an odds ratio of 3.17. Additionally, patients who received bicarbonate had a higher risk of developing prolonged hospital stay and dialysis dependency, as well as an increased mortality rate. Moreover, the use of sodium bicarbonate was found to be a risk factor for dialysis and a predictor of mortality. When subgrouping patients based on the cause of their rhabdomyolysis, Kim et al. found that sodium bicarbonate is associated with developing AKI in patients with both medical and surgical causes of rhabdomyolysis. Of interest, the association was stronger in patients with surgical causes of rhabdomyolysis. Regarding the volume of the fluids received during the first 72 hours of treatment, higher mortality rates were associated with receiving more than 5.5 mL/kg/h of fluids. The treatment with both high-volume fluid and sodium bicarbonate was associated with a higher risk of AKI more than the exposure to one risk factor [108].

The study by Kim et al. shows that sodium bicarbonate not only lacks benefits, but is also associated with worse outcomes in patients with rhabdomyolysis [108,109]. The worse outcomes associated with sodium bicarbonate could be due to the bicarbonate-induced paradoxical respiratory acidosis, which may increase the intracellular acidosis, especially in the absence of effective ventilation to wash out the bicarbonate-induced production of CO_2_. Moreover, bicarbonate’s negative effect on the oxygen delivery, as discussed earlier, may affect the oxygen delivery to renal tubules [110]. Given the aforementioned data, we recommend against the routine use of sodium bicarbonate in patients with rhabdomyolysis, unless in cases of concomitant severe metabolic acidosis.

##### Sodium Bicarbonate in Patients with Lactic Acidosis

Lactic acidosis results from increased lactic acid production or decreased clearance. According to Cohen and Woods’ classification of lactic acidosis, lactic acidosis can be divided into two main categories: (1) lactic acidosis type A, which is associated with decreased tissue oxygenation, and (2) lactic acidosis type B, which exists in the absence of tissue hypoxia. Lactic acidosis type B is further subdivided into (1) lactic acidosis type B1, which results from underlying diseases such as sepsis, liver failure, pheochromocytoma, malignancy, and thiamine deficiency, (2) lactic acidosis type B2, which is induced by toxins or drugs such as epinephrine, inhaled nitric oxide, terbutaline, ethylene glycol, methanol, ethanol, propofol, linezolid, isoniazid, nitroprusside, acetaminophen, and aspirin, and (3) lactic acidosis type B3, which is caused by inborn errors of metabolism, such as pyruvate dehydrogenase deficiency, glucose-6-phosphatase deficiency, fructose-1,6-diphosphatase deficiency, and oxidative phosphorylation defects in the mitochondria [111,112].

Sepsis can increase lactic acid production through tissue hypoxia and decrease lactic acid clearance through pre-existing or sepsis-induced liver dysfunction, as well as through the administration of catecholamines. Consequently, sepsis can cause both lactic acidosis type A and type B [111]. Interestingly, beta-blockers were found to reduce the production of lactic acid in septic patients. This may explain the lower mortality associated with using beta-blockers in septic patients [111,113]. The use of sodium bicarbonate in septic patients with lactic acidosis has been a controversial topic among clinicians for decades because of its potential harm and questionable benefit [114].

Many studies have demonstrated that the use of sodium bicarbonate in septic patients with lactic acidosis does not result in improved mortality outcomes. In a retrospective study by Zhang et al. in 2018, there was no significant association observed between mortality and sodium bicarbonate infusion in septic patients with metabolic acidosis and elevated lactic acid levels [115]. In another retrospective study by El-Solh in 2010, sodium bicarbonate continuous infusion in septic patients with high lactate levels of 4.87 ± 2.1 mmol/L and pH of 7.17 ± 0.1 was associated with decreased ventilation duration and reduced ICU stay compared to the control group with lactate levels of 3.36 ± 1.7 mmol/L and pH of 7.19 ± 0.09 [116]. However, no statistically significant association was found between sodium bicarbonate infusion and 28-day mortality [116]. These findings suggest that sodium bicarbonate administration may have potential benefits in decreasing ventilation duration and ICU stay, but does not impact overall mortality.

Of interest, in a retrospective study by Kim et al. in 2013, sodium bicarbonate was associated with a high mortality rate in patients with lactic acidosis. This outcome might be attributed to the fact that patients receiving bicarbonate therapy often present with higher initial lactic acid levels and more severe acidosis. However, even after excluding patients with less severe acidosis, the observed significant association remained unaffected [117]. In a meta-analysis of two randomized trials and three observational studies by Lo et al. in 2019, the use of sodium bicarbonate was not significantly associated with improved mortality in septic patients with HAGMA [77,82,116,117,118,119].

The Surviving Sepsis Campaign guidelines in 2021 suggest against the use of sodium bicarbonate to reduce vasopressor requirements or to improve hemodynamics in patients with septic shock and lactic acidosis; this is a weak recommendation with a low quality of evidence [3]. In contrast, they suggest the use of sodium bicarbonate in septic patients with severe acidosis (pH ≤ 7.2) and AKI stage 2 and 3 as a weak recommendation with a low quality of evidence [3]. Similarly, the guidelines by the French Intensive Care Society and the French Emergency Medicine Society recommend the use of sodium bicarbonate in ICU patients with moderate to severe AKI and severe metabolic acidosis (pH  ≤  7.20, PaCO_2_  <  45 mmHg) as an optional recommendation with a moderate level of evidence [1].

#### 2.5.2. Sodium Bicarbonate in Patients with Normal Anion Gap Metabolic Acidosis (NAGMA)

NAGMA happens because of sodium bicarbonate loss through the gastrointestinal tract or the kidneys. It may also happen due to hyperchloremia and impaired ammonia excretion [84,120]. Gastrointestinal bicarbonate loss is commonly caused by severe diarrhea, ileostomy, and pancreatic fistulas. Additionally, cholestyramine may also cause gastrointestinal bicarbonate loss. Moreover, urinary diversion may lead to bicarbonate loss due to prolonged exposure of urine to gastrointestinal mucosa [121,122,123].

About 4500 mmol of bicarbonate is filtered through renal glomeruli. Almost all the filtered bicarbonate is reabsorbed through the renal tubules, and none appears in the urine. About 80% of the filtered bicarbonate is reabsorbed in the proximal tubules, while the remaining bicarbonate is reabsorbed through the ascending limb of the loop of Henle, distal tubules, and collecting ducts [120,124]. Loss of bicarbonate through the kidneys may happen due to decreased bicarbonate reabsorption in the proximal tubules, as seen in renal tubular acidosis (RTA) type 2. This condition may arise from high levels of excreted monoclonal immunoglobulin light chains in monoclonal gammopathies and medications, such as ifosfamide, acetazolamide, topiramate, valproic acid, and various antiretrovirals [125,126].

Impaired renal excretion of protons in the distal tubules can affect bicarbonate production by carbonic anhydrase in type A intercalated cells. This condition is known as RTA type 1, which could be caused by many autoimmune diseases and medications such as foscarnet, lithium ifosfamide, and amphotericin B [121,127]. Inadequate sodium reabsorption in the collecting ducts, as in RTA type 4, may occur due to aldosterone insensitivity and medications such as aldosterone antagonists, angiotensin-converting enzyme inhibitors, nonsteroidal anti-inflammatory drugs, calcineurin inhibitors, and angiotensin II receptor blockers [128,129]. Moreover, hyperchloremia induced by normal saline can impair bicarbonate reabsorption. A similar effect was also observed with other chloride-rich fluids, such as total parenteral nutrition solutions [120].

In contrast to HAGMA, there are no clinical trials assessing the effectiveness of sodium bicarbonate administration in NAGMA. Treating critical NAGMA with sodium bicarbonate is based on understanding the pathophysiology of developing NAGMA [4]. According to the guidelines by the French Intensive Care Society and the French Emergency Medicine Society, sodium bicarbonate is recommended for gastrointestinal or renal bicarbonate loss in patients with poor clinical tolerance [1].

### 2.6. Sodium Bicarbonate in Acidotic Patients with Acute Kidney Injury (AKI)

Acute kidney injury (AKI) is defined as a sudden and often reversible decline in kidney function that presents as a rapid increase in the levels of serum creatinine, an acute decrease in the urine output, or both. AKI can be classified based on the etiology into prerenal, renal, and postrenal azotemia [130]. Acidosis develops in patients with AKI due to increased excretion of sodium bicarbonate, decreased bicarbonate synthesis, hyperphosphatemia, hyperchloremia, and retention of unmeasured anions, such as sulfate, acetate, and citrate [131]. Acute acidosis is the most common acid–base disturbance in patients with AKI. The co-existence of acute acidosis and AKI is an indicator of worse outcomes and increased mortality [132].

In a retrospective study by Zhang et al., there was no significant association observed between mortality and sodium bicarbonate infusion in septic patients with metabolic acidosis. However, sodium bicarbonate was associated with decreased mortality in septic patients with AKI stages 2 and 3 [115]. In a clinical trial by Jaber et al., treatment with sodium bicarbonate was found to be associated with a decreased 28-day mortality and lower organ failure by day 7 in acidotic patients with AKI network (AKIN) scores of 2 or 3 [79,82]. In the study, no information was provided about the portion of saline used in the study population. Saline is commonly administrated in patients with AKI and may cause hyperchloremic metabolic acidosis. The use of other types of fluids could diminish the need for sodium bicarbonate and affect the acidosis incidence. One of the other limitations in this study is that many other factors, such as hemodynamic stability, might affect the incidence of organ failure [133].

In contrast to the previous clinical trial, a retrospective study in 2021 by Fujii et al. showed that the reduction in the ICU mortality was not statistically significant in acidotic patients with AKI treated with sodium bicarbonate compared to those who did not receive bicarbonate [78]. This contradiction between the two studies might be attributed to the difference in bicarbonate dosage, study design, and sample size between the two studies. Also, it is worth to mention that patients in the study by Fujii et al. received only 110 mmol of sodium bicarbonate, while the patients in the clinical trial by Jaber et al. received 250 mmol of sodium bicarbonate in the first 24 h [78,82].

Subgrouping acidotic patients with AKI based on the AKI etiology, severity of acidosis, etiology of acidosis, severity of AKI, hemodynamic status, and concurrent comorbidities is important, as all these variables may affect the response to sodium bicarbonate. Zhang et al. found that there is no significant association between sodium bicarbonate administration and mortality in the overall population of acidotic patients with AKI. By subgrouping the patients based on AKI-associated co-morbidity, Zhang et al. found that sodium bicarbonate administration is associated with lower mortality in AKI patients with pancreatitis with a hazard ratio of 0.53 and severe acidosis (pH < 7.15) with a hazard ratio of 0.75 [134]. Similarly, a recent study in 2023 by Wang et al. showed that sodium bicarbonate administration was not associated with decreased mortality in patients with AKI complicated with metabolic acidosis. However, after subgrouping the patients based on the anion gap, they found that improved mortality was associated with sodium bicarbonate administration in AKI patients with high-anion gap metabolic acidosis (anion gap > 18 mmol/L) [135]. More clinical trials are encouraged to evaluate the effectiveness of sodium bicarbonate in the different subgroups of acidotic patients with AKI.

According to the guidelines by the French Intensive Care Society and the French Emergency Medicine Society, sodium bicarbonate should be administered in ICU patients with moderate to severe AKI and severe metabolic acidosis (pH  ≤  7.20, PaCO_2_  <  45 mmHg) as an optional recommendation with a moderate level of evidence [1]. Moreover, the Surviving Sepsis Campaign guidelines in 2021 suggest the use of sodium bicarbonate in septic patients with severe acidosis (pH ≤ 7.2) and AKI stages 2 and 3 as a weak recommendation with a low quality of evidence [3].

### 2.7. Sodium Bicarbonate in Cardiac Arrest Resuscitation

During cardiac arrest, the resultant hypoxia is expected to cause high levels of lactic acid, and as a result, develops acidosis. Theoretically, patients with cardiac arrest may benefit from sodium bicarbonate administration, as it corrects the patient’s potential acidosis and increases the effectiveness of resuscitation medications, such as epinephrine. However, sodium bicarbonate administration may cause decreased oxygen delivery, as mentioned above, and increase arterial PCO_2_, leading to paradoxical respiratory acidosis, especially in those patients with cardiac arrest who do not have enough respiratory drive to wash out the CO_2_ produced by bicarbonate administration. Sodium bicarbonate administration may also increase the risk of developing acute hypernatremia, hyperosmolarity, hypervolemia, alkalemia, hypokalemia, hypocalcemia, and intracellular acidosis [7,14,136,137]. As a result of having these potential benefits and harmful effects, an ongoing debate about the use of sodium bicarbonate during cardiac arrest resuscitation has continued.

Given that there is no evidence of its potential benefit during resuscitation, as discussed in detail below, the theoretical benefit and harm of sodium bicarbonate created a historical and ongoing debate about its benefit in resuscitation. Since the early American Heart Association (AHA) recommendations in 1976 that supported the use of sodium bicarbonate in cardiac arrest resuscitation, a debate raised due to concerns about the potentially harmful effects of sodium bicarbonate. Since 2010, the advanced cardiac life support (ACLS) guidelines suggest against the routine use of sodium bicarbonate during cardiac arrest resuscitation [138,139,140]. The AHA recommends the use of sodium bicarbonate during ACLS for only limited indications, including severe acidosis (pH < 7.2), hyperkalemia (potassium level > 5.5 mmol/L), and tricyclic antidepressant toxicity. Moreover, according to the current guidelines by the European Resuscitation Council (ERC), sodium bicarbonate is not recommended routinely during cardiac arrest resuscitation because of the lack of evidence that supports its usage. The ERC guidelines suggest the use of sodium bicarbonate in an unresponsive prolonged resuscitation in presence of effective ventilation to offset the effect of the expected acidosis in prolonged resuscitation [138,141]. This indicates that according to current guidelines, the routine administration of sodium bicarbonate empirically during cardiac arrest resuscitation is not recommended.

#### 2.7.1. Sodium Bicarbonate in Out-of-Hospital Cardiac Arrest (OHCA)

In patients with OHCA, Kawano et al. found that prehospital administration of sodium bicarbonate by paramedics was negatively associated with neurological outcomes to hospital discharge and survival rate. After subgrouping the patients based on the length of resuscitation, the dose of epinephrine, and the initial rhythm, they found a strong negative association between sodium bicarbonate administration and smaller doses of epinephrine, longer duration of resuscitation, and shockable rhythm. There was no positive association found between sodium bicarbonate administration and any of the subgroups [14]. In a systematic review by Alshahrani et al., a meta-analysis of four observational studies and a randomized clinical trial showed no statistically significant difference in the return of spontaneous circulation (ROSC) rate between the sodium bicarbonate group and control [14,142,143,144,145,146]. Moreover, in the same study, a meta-analysis of three observational studies and four randomized clinical trials showed no significant difference in the survival rate after discharge from hospital between the sodium bicarbonate group and the control group [14,142,143,145,146,147,148,149]. In another recent systematic review and meta-analysis by Xu et al., sodium bicarbonate administration was found not to be associated with short-term or long-term survival rates. Of interest, they found that sodium bicarbonate administration may worsen long-term survival [150].

##### Sodium Bicarbonate in Out-of-Hospital Cardiac Arrest (OHCA) Based on the Presenting Rhythm

Subgrouping patients based on the presenting rhythm is important as different types of rhythm during cardiac arrest may affect the response to sodium bicarbonate during resuscitation. In a retrospective study by Niederberger et al., bicarbonate administration was associated with higher ROSC rates in patients with asystole as the presenting rhythm compared to control. Moreover, survival rates were found to be higher in sodium bicarbonate group in patients with asystole or pulseless electrical activity (PEA) compared to control. Of interest, no significant difference was noticed between the sodium bicarbonate group and control in patients with presenting rhythms of ventricular fibrillation (VF) or ventricular tachycardia (VT) [151,152,153]. This study indicates that sodium bicarbonate administration could be beneficial in non-shockable rhythms. Prospective clinical trials are encouraged to test the effect of sodium bicarbonate subgroups of different rhythms.

##### Sodium Bicarbonate in Out-of-Hospital Cardiac Arrest (OHCA) Based on the Continent

In a meta-analysis of three studies of the Asian population, Wu et al. found a higher ROSC rate in the sodium bicarbonate group compared to patients who did not receive sodium bicarbonate during resuscitation with an odds ratio of 2.335 [145,146,154,155]. However, this association is not statistically significant [155]. In the same study, a meta-analysis of two studies of the North American population showed that a significantly negative association between sodium bicarbonate administration and the ROSC rate with an odds ratio of 0.521 [14,155,156]. Despite the absence of a significant association between sodium bicarbonate administration and ROSC rate in the Asian population, the ROSC rate was seen more in the sodium bicarbonate group compared to the North American group. The cause of this difference is still unclear, and further clinical studies are encouraged [155]. In the meta-analysis of the all six studies included in the systematic review, no significant association was found between sodium bicarbonate administration and both the ROSC rate and the survival-to-discharge rate [14,145,146,154,155,156,157]. The results of this meta-analysis support the current recommendations by the AHA against the routine use of sodium bicarbonate during resuscitation.

In a randomized clinical trial by Touron et al., there was no significant association between sodium bicarbonate administration and the neurologic outcomes in the French population. In contrast, prehospital administration of sodium bicarbonate was significantly associated with worse neurologic outcomes in the North American population compared to control [15]. The two studies by Touron et al. and Wu et al. show that the North American population may have worse outcomes associated with sodium bicarbonate administration during cardiac arrest resuscitation compared to other populations [15,155]. Clinical trials are encouraged to investigate the effect of sodium bicarbonate administration on the ROSC rate and functional outcomes after ROSC in different races. Genetic factors may contribute to the different responses to sodium bicarbonate in the different continents.

#### 2.7.2. Sodium Bicarbonate in In-Hospital Cardiac Arrest (IHCA)

In a retrospective study by Benz et al., sodium bicarbonate administration was significantly associated with a lower ROSC rate with an odds ratio of 0.35 and lower survival to discharge with an odds ratio of 0.27 in patients with IHCA [158]. In another retrospective study by Zaidi et al., sodium bicarbonate administration was found to be significantly associated with the reduction in both the neurological recovery at 30 days and the odds of discharge in patients with IHCA [16]. These studies support the recommendations by AHA against the routine use of sodium bicarbonate during cardiac arrest resuscitation. It is noticeable that most of the studies which discussed the use of sodium bicarbonate in patients with OHCA or IHCA did not subgroup the patients based on the initial baseline pH, presence of underlying cardiac disease, the patient’s race, the initial potassium level, the presenting rhythm, and the presence of reversible causes of cardiac arrest, such as hypothermia, pulmonary embolism, and electrolyte disturbance. Further clinical studies with larger populations, stratified based on the previously mentioned characteristics, are encouraged.

#### 2.7.3. Sodium Bicarbonate in Acidotic Patients with Cardiac Arrest

Theoretically, correcting acidosis during cardiac arrest resuscitation may be beneficial, as it may offset the acidosis-induced cardiomyopathy, adrenergic receptors insensitivity, and metabolic disturbances. In a randomized clinical trial by Ahn et al., sodium bicarbonate administration was significantly associated with increasing pH and bicarbonate levels in patients with cardiac arrest and severe acidosis (pH < 7.1 or bicarbonate < 10 mEq/L) compared to those who received normal saline. However, there was no significant association between sodium bicarbonate group and normal saline group in terms of better neurologic survival at 1 month or sustained ROSC [143]. The main limitation of this study is the small sample size of only 50 patients.

According to the AHA, sodium bicarbonate is not recommended routinely during resuscitation. Despite absence of a strong evidence, AHA recommends the use of sodium bicarbonate during ACLS in patients with severe acidosis (pH < 7.2) [138,140]. More clinical trials are encouraged to investigate the role of sodium bicarbonate in acidotic patients with cardiac arrest.

### 2.8. Sodium Bicarbonate in Hyperkalemic Patients

Hyperkalemia can cause depolarization in myocardial membranes, leading to electrocardiogram (ECG) abnormalities, such as peaked T waves, long PR intervals, wide QRS complexes, and sine-wave patterns. If left untreated, these changes can progress, potentially leading to cardiac arrest. Several treatments can be used to treat hyperkalemia, including intravenous calcium salts, insulin with glucose, albuterol, and intravenous sodium bicarbonate [159,160,161,162].

In patients with severe hyperkalemia, sodium bicarbonate and calcium salts are used routinely. Calcium salts stabilize cardiac cellular membranes and sodium bicarbonate helps with potassium redistribution into cells via hydrogen and potassium exchange through blood alkalinization [159,160]. Despite the routine use of sodium bicarbonate in the treatment of hyperkalemia, no strong evidence supports this practice [160,163,164]. Many studies discussed the use of continuous infusion of sodium bicarbonate in the treatment of hyperkalemia. However, in routine clinical practice, sodium bicarbonate is typically administered as a bolus. This distinction suggests that the findings from these studies may not be directly applicable to or supportive of the use of sodium bicarbonate in bolus form [164].

In 2021, a study by Geng et al. showed that adding IV sodium bicarbonate to IV insulin in the treatment of patients with hyperkalemia was not significantly associated with lowering potassium levels [164,165]. However, Gen et al. did not mention the baseline pH for all patients or stratify patients based on their baseline pH. The baseline pH and the type of acidosis may manipulate the sodium bicarbonate effects, also. Sodium bicarbonate effects may also be influenced by the cause of hyperkalemia and the concomitant administration of insulin [164,165]. Although sodium bicarbonate treatment is mainly based on understanding the pathophysiology and there is a lack of evidence of its efficacy in the treatment of stable patients with hyperkalemia, many studies have shown a promising role for sodium bicarbonate administration in hyperkalemic patients with cardiac arrest, as mentioned below.

Wang et al. found that sodium bicarbonate administration is significantly associated with sustained ROSC in hyperkalemic patients with IHCA [166]. They also found a positive correlation between serum potassium levels and the efficacy of calcium and sodium bicarbonate administration [166]. This study supports the AHA recommendations of sodium bicarbonate administration in hyperkalemic patients with cardiac arrest [138,140].

In contrast to IHCA, Lee et al. found that hyperkalemic patients with OHCA who received anti-hyperkalemic medications, such as sodium bicarbonate, calcium, and insulin with glucose, had a lower rate of ROSC compared to those who did not receive anti-hyperkalemic medications [161]. On subgrouping the patients according to initial potassium levels, there was no statistically significant difference between the group that received anti-hyperkalemic medications and the group that did not receive any anti-hyperkalemic medications [161]. This study demonstrates that administration of anti-hyperkalemic agents, such as sodium bicarbonate, calcium, and insulin with glucose, does not affect the rate of ROSC in OHCA. The results of this study are limited by many factors, such as having a small sample size, inability to identify the timing of anti-hyperkalemic drug administration, and absence of baseline pH at the time of anti-hyperkalemic drug administration. More clinical trials are encouraged to investigate the role of sodium bicarbonate in the treatment of hyperkalemia in both stable patients and patients with cardiac arrest.

### 2.9. Sodium Bicarbonate in Acidosis-Induced Coagulopathy

In an animal study, both acidosis and high lactic acid are significantly associated with increased postoperative bleeding in pigs with cardiac surgery [167]. The pathophysiology of acidosis-induced coagulopathy is not fully understood. Theoretically, acidosis may affect the efficacy of the enzymes controlling the activation of the coagulation factors, downregulate the production of some coagulation factors, or impair the platelets’ aggregation or adhesion. In animal studies, acidosis was found to affect coagulation in different ways [8,168,169,170].

Martini et al. found that acidosis did not affect fibrinogen production. However, it increased its consumption, leading to fibrinogen deficiency in acidotic pigs [171]. Acidosis was also associated with decreased platelet counts, prolonged partial activated thromboplastin time (PTT), and prolonged prothrombin time (PT) [171]. This indicates that acidosis may induce disseminated intravascular coagulation (DIC)-like changes in the coagulation parameters. In accordance with this study, Broersma et al. found an association between lower pH values and the development of DIC through thrombi formation in different organs and consumption of coagulation factors in acidotic dogs [172]. Moreover, the activity of many coagulation factors was found to be negatively affected by acidosis [173,174]. Acidosis was also found to suppress platelet function [175,176].

Acute correction of acidosis may appear to be advantageous to reverse the acidosis-induced coagulopathy. Nevertheless, animal studies did not show a significant benefit of acute administration of alkalinizing agents, such as sodium bicarbonate and THAM to reverse coagulopathy immediately [177,178,179]. However, in a recent in vitro study, reversal of acidosis by sodium bicarbonate was found to be associated with improvement in some coagulation parameters [180]. No clinical trials or in vivo observational studies in humans studied the effect of acidosis reversal on coagulation recovery. In vivo clinical studies in humans are encouraged to evaluate the effects of sodium bicarbonate administration on coagulation parameters and platelet function in acidotic patients with acidosis-induced coagulopathy.

## 3. Conclusions

Despite the guidelines and restrictions set by professional medical societies, sodium bicarbonate has been overused in clinical practice to treat a variety of disorders, such as acute metabolic acidosis, cardiac arrest, rhabdomyolysis, DKA, AKI, hyperkalemia, and adrenergic receptors’ resistance to catecholamine in patients with shock. The overuse of sodium bicarbonate could be because of the desire to correct the ABG parameters rapidly instead of achieving homeostasis by treating the cause of metabolic acidosis. It is crucial to pay attention to the potential harm that could be caused by excessive sodium bicarbonate administration.

The evidence supporting the use of sodium bicarbonate in these different pathologies varies significantly. More clinical trials are encouraged to evaluate the potential benefit and harm of sodium bicarbonate administration in patients with different pathologies.
